# IGF-I and IGFBP-1 in Relation to Body Composition and Physical Performance in Female Olympic Athletes

**DOI:** 10.3389/fendo.2021.708421

**Published:** 2021-08-16

**Authors:** Emma Eklund, Anton Hellberg, Bo Berglund, Kerstin Brismar, Angelica Lindén Hirschberg

**Affiliations:** ^1^Department of Women’s and Children’s Health, Division of Neonatology, Obstetrics and Gynecology, Karolinska Institutet, Stockholm, Sweden; ^2^Department of Internal Medicine, Karolinska Institutet, Karolinska University Hospital, Stockholm, Sweden; ^3^Department of Molecular Medicine and Surgery, Karolinska Institutet, Karolinska University Hospital, Stockholm, Sweden; ^4^Department of Gynecology and Reproductive Medicine, Karolinska University Hospital, Stockholm, Sweden

**Keywords:** athletic performance, body composition, female athlete, IGF-I - insulinlike growth factor 1, GFBP-1 - insulin-like growth factor binding protein 1

## Abstract

**Introduction:**

Insulin- like growth factor-I (IGF-I) is an anabolic hormone that may affect athletic performance in female athletes, and insulin-like growth factor binding protein-1 (IGFBP-1) is an important regulator of bioactive IGF-I. There is limited knowledge of the role of endogenous IGF-I and IGFBP-1 for body composition and physical performance in female elite athletes.

**Purpose:**

To examine IGF-I, age adjusted IGF-I (IGFSD), IGFBP-1 and insulin in female Olympic athletes compared with controls and different sport categories, and in relation to body composition and physical performance in the athletes.

**Methods:**

Female athletes (n=103) and untrained controls (n=113) were included in this cross-sectional study. Body composition was established by dual-energy X-ray absorptiometry. Serum IGF-I and IGFBP-1 were analyzed by radioimmunoassay and IGFSD was calculated. Insulin was analyzed by electrochemiluminescence immunoassay. Athletes were offered to participate in standardized physical fitness tests.

**Results:**

The athletes demonstrated significantly higher IGF-I, IGFSD and IGFBP-1 and lower insulin levels than controls (p<0.05, p<0.05, p<0.01, p<0.001 respectively). Power athletes had significantly higher IGFSD compared to both endurance and technical athletes (p<0.05, p<0.01, respectively). In athletes and controls combined, significant positive correlations were found between IGF variables and higher bone mineral density (BMD) and lean mass and lower fat percent. IGF-I was positively correlated with squat jump (r_s_ = 0.28, p<0.05) and IGFBP-1 correlated positively with squats (r_s_ =0.35, p<0.05).

**Conclusion:**

We found higher IGF-I, IGFSD and IGFBP-1 in female athletes than controls, and the highest IGFSD in power athletes. IGF-I and IGFBP-1 were related to increased BMD and lean mass and lower fat percent, as well as were positively associated with physical fitness tests. Future studies are needed to elucidate if these results reflect adaptive responses to physical activity or genetic predisposition.

## Introduction

Insulin-like growth factor-I (IGF-I) is considered an anabolic hormone with potential performance enhancing effects. IGF-I production is under stimulation of growth hormone (GH) secreted from the pituitary gland (1). The majority of circulating IGF-I is bound to IGF binding proteins (IGFBPs) 1-6, which regulate IGF-I bioavailability, as well as having IGF independent effects (2). In adult patients with growth hormone deficiency, GH supplementation, and thereby increased IGF-I, results in reduced fat mass, increased lean body mass, maximal oxygen uptake and power output (3–5). However, it is questioned if GH/IGF-I supplementation exerts similar performance enhancing effects in healthy subjects, as well as in athletes (3, 6–8). Nevertheless, GH and IGF-I are used as doping agents among athletes (3, 9, 10), this despite being banned by the World Anti-Doping agency (6).

IGBP-1 is an important regulator of free IGF-I (1, 2, 11). Circulating IGFBP-1, mainly produced in the liver, is regulated by insulin at transcriptional level (2) and induced by hypoxia inducible factor 1-alpha (HIF-1a) during hypoxia (12, 13). High IGFBP-1 levels are associated with low insulin secretion and increased insulin sensitivity but also with increased GH levels (2, 14). IGFBP-1 binds independent of IGF-I to an integrin receptor through which it stimulates vascular nitric oxide synthesis, and thus vasodilatation and blood flow. Extensive exercise is associated with hypoxic reaction and increased HIF-1a (15) and IGFBP-1 serum levels (1).

Most previous studies have focused on potential anabolic properties of exogenous GH and IGF-I (3, 8, 16, 17) whereas, there is limited knowledge of the role of endogenous IGF-I, especially in female elite athletes. The few previous studies investigating IGF-I levels in female athletes and controls have shown conflicting results, demonstrating both higher levels in athletes (18, 19) or no significant difference between groups (20, 21). Most of these studies have included a small number of participants (18, 20, 21), with the exception of a large study by Healy et al. (19) investigating post-competition IGF-I levels in elite athletes in comparison with resting levels of untrained healthy controls. Furthermore, varying endogenous IGF-I levels in female athletes of different types of sports have been reported (20, 22). Although IGF-I is known to decrease with age (14, 23), age adjusted IGF-I has not been reported in the majority of studies (18, 20, 21).

In adult female elite athletes, small studies have demonstrated a positive association between endogenous IGF-I and bone mineral density (BMD) (20), as well as body weight (24). In untrained women, IGFBP-2 has been associated with peak oxygen uptake (25), and in adolescent girls, IGF-I was positively related to muscle volume, whereas no significant relation was found to aerobic performance (26). Furthermore, in a large population of healthy young and older women, serum IGF-I was associated with peak exercise capacity (27). However, to our knowledge, no previous study has evaluated endogenous IGF-I and IGFBP-1 in relation to body composition and physical fitness tests in female elite athletes.

To provide a better understanding of the role of endogenous IGF-I and IGFBP-1 for body composition and physical fitness in female athletes, we compared serum levels of unadjusted IGF-I, age adjusted IGF-I (IGFSD), insulin and IGFBP-1 in female Olympic athletes with untrained controls and between sport categories. Furthermore, we explored possible associations between these levels, body composition and physical fitness tests. We hypothesized the findings of higher IGF-I and IGFBP-1 levels in the athletes, and positive associations to muscle mass, bone mass and physical fitness tests.

## Materials and Methods

### Data Collection and Study Population

The athletes participating in this study, were part of a study cohort including Swedish Female Olympic athletes (n=106), members of a Swedish Olympic team or part of the high-performance programs of the Swedish Olympic Committee (SOC). The recruitment was performed between 2011-2015, in order to obtain a representative population of female Swedish Olympic athletes for the summer or winter Olympic games during this period (28, 29). The athletes were divided into sport categories, Power, Endurance and Technical, depending on type of sport (**Table 1**) as previously described (30). All athletes were screened for anabolic androgenic steroids at the Doping Laboratory, Karolinska University Hospital, Huddinge, none demonstrating atypical findings.

**Table 1:** Sport category and type of sport for the female Olympic athletes.

Sport Category	Type of sport
Power (n = 62)	Gymnastics, long jump, alpine skiing, figure skating, ice hockey, judo, handball, soccer, high jump, pole vault, taekwondo, fencing, tennis, boxing.
Endurance (n = 29)	Cycling, canoeing, long distance running, marathon running, triathlon, biathlon, cross country skiing, swimming, mountain bike, rowing.
Technical (n = 12)	Dressage, curling, shooting sport, sailing.

In addition, healthy, age- and body mass index (BMI) matched female controls (n=117), having a physical activity of maximum 2 hours per week and no prior participation in elite level sports were included in the original study cohort. A more detailed description of the study cohort has been published previously (28). The subjects were investigated at the Women’s Health Research Unit, Karolinska University Hospital or in connection with training camps. Data on health status and physical activity per week were collected by questionnaire for all participants. In addition, gynecological data (hormonal contraceptive use, bleeding pattern, menstrual disorders and pregnancies) were obtained. Participants with amenorrhea (absence of menstruation during at least the previous 3 months) or oligomenorrhea (5-9 periods during the past year, occurring at interval > 6 weeks) were defined as having menstrual dysfunction. Data on sports discipline and training debut was collected from the athletes.

All participants were in general good health. A few had well-controlled treated hypothyroidism, five athletes and three controls. One female Olympic athlete had previously been diagnosed with diabetes mellitus type 1 (DM type 1) and was medically well controlled. She was excluded from some of the analyses, see *Statistical Analyses*.

The present study population included 103 female Olympic athletes and 113 controls, from which sufficient blood samples were available for analyses of IGF-I and IGFBP-1. A fasting blood sample was collected at rest (no exercise at least 12 h prior) between 07.00 and 10.00 hour and stored at -20 C until further analysis. Blood samples were collected randomly according to the menstrual cycle. Body composition was determined by dual-energy X-ray absorptiometry (DXA) and a significant number of the athletes performed standardized physical fitness tests *via* SOC.

The study was approved by the Regional Ethics Committee, Stockholm (EPN 2011/1426-32) and written informed consent was given by all participants.

### Body Composition

Body composition, including bone mineral density (BMD), fat mass and lean mass, was established for 161 study participants (64 athletes and 97 controls) by dual energy X-ray absorptiometry (DXA), Lunar Prodigy Advance (GE, Healthcare, Madison, Wisconsin, USA) at the Karolinska University Hospital. Spinal BMD was determined from the whole body DXA. Z-scores were estimated from the mean BMD and their SD values supplied by the manufacturer of the scanner (Z-score < - 2SD being defined as low BMD), as previously described (28).

### Physical Fitness Tests

Athletes were offered to participate in standardized physical fitness tests, part of the “Physical Profile” *via* SOC at the Sports Institute, Bosön, Stockholm. Mainly power athletes participated in the physical fitness tests. All athletes did not participate in all tests, and only athletes where IGF variables had been measured were included in the physical fitness test analyses. Physical fitness tests included 3000 m running (n=20), bench press (n=44), chins (n=49), squats (n=50), squat jump (SJ) (n=56) and countermovement jump (CMJ) (n=57). SJ and CMJ are validated tests for measuring explosive power of the lower limbs. Maximum height (cm) was recorded using an infrared contact plate, IVAR equipment (IVAR Ltd, Tallin, Estonia) (31). 3000 m reflects aerobic performance and bench press, squats and chins measure strength (32).

### Endocrine Analyses

Serum levels of IGF-I were determined by an in-house radioimmunoassay (RIA) after separation from IGFBPs by acid ethanol extraction and cryoprecipitation. To minimize interference of remaining IGFBPs, des (1-3) IGF-I was used as radio-ligand (33). The intra-and inter-assay CV were 4% and 11%, respectively and detection limit 6 µg/L. The concentrations of fasting IGFBP-1 in serum were determined by an in-house RIA according to Póvoa et al. (34). The sensitivity of the RIA was 3 µg/l and the intra- and inter-assay CV were 3% and 10%, respectively. In healthy adults a linear inverse correlation, with no gender differences, exists between logarithmic transformed IGF-I levels and age. To correctly compare IGF-I levels between subjects of different age, IGF-I values were also expressed as SD scores (IGFSD) calculated from the regression of the IGF-I values of healthy adult subjects aged 20-95 years (14, 35, 36). Fasting levels of insulin were measured using the clinical standard method electrochemiluminescence immunoassay (ECLIA) [Roche Cobas 8000 (e602)] at the Karolinska University Laboratory, with a reference range (according to the manufacturer Roche) of 2.0-25. mIE/L. Fasting serum insulin between 14-25 mIE/L indicates insulin resistance.

### Statistical Analyses

Statistical analyses were performed using Statistica version 13 [TIBCO Software inc (2018)]. Continuous data was presented as mean ± SD or as median and interquartile range (25th-75th percentile) depending on distribution. IGFBP-1 (all groups) and insulin (for controls only) were not normally distributed and were therefore square root transformed (IGFBP-1) or log transformed (insulin) prior to parametric statistical tests. For comparison of anthropometric data, body composition, IGF-I, IGFSD, IGFBP-1 and insulin between athletes and controls, the student’s t-test was applied. In comparisons of categorical data including hormonal contraceptive use, menstrual dysfunction and frequency of fasting insulin indicative of insulin resistance between the groups, the Pearson Chi-square or Fisher´s exact test was used. When comparing anthropometric data, body composition, insulin levels, IGF-I, IGFSD and IGFBP-1 between sport categories one-way analysis of variance (ANOVA) was used, followed by pairwise comparison between groups (LSD test). To study whether the differences between groups, regarding IGF-I, IGFSD and IGFBP-1, were due to hormonal contraceptive use, two-way ANOVA was applied. Spearman correlation was used to evaluate association between variables. The athlete with DM type 1 was excluded from the analyses of IGF-I, IGFSD, IGFBP-1 and insulin. A forward stepwise multiple regression analysis was performed to evaluate the variance in IGF-I levels. P-values <0.05 was considered statistically significant.

## Results

### Demographic Characteristics and Body Composition

**Table 2** shows general characteristics and body composition of the female athletes and controls. The athletes were significantly taller and had a higher body weight but comparable BMI to the controls (**Table 2**). Furthermore, the athletes had significantly higher BMD and lean mass and lower fat percent compared to controls, as previously published in our larger cohort (28). Hormonal contraceptive use was comparable between groups, whereas menstrual dysfunction was significantly more common among the athletes than the controls (**Table 2**).

**Table 2:** General characteristics and body composition in female Olympic athletes and controls.

Parameter	Controls	Athletes
n	113	103
Age	26.1 ± 5.5	25.7 ± 5.3
BMI	22.0 ± 2.6	22.0 ± 1.9
Weight (kg)	62.0 ± 8.4	64.7 ± 7.3*
Height (m)	1.68 ± 0.07	1.71 ± 0.06***
HC use, n (%)	45 (39.8)	40 (38.8)
MD, n (%)	3 (5)	15 (24)**
**Body composition**		
n	97	64
Total BMD (g/cm^2^)	1.15 ± 0.07	1.25 ± 0.08***
Spinal BMD (g/cm^2^)	1.01 ± 0.10	1.11 ± 0.11***
Z-score	0.35 ± 0.86	1.63 ± 1.01***
Body fat (%)	31.8 ± 6.6	18.5 ± 6.0***
Lean mass total (kg)	40.4 ± 4.1	50.0 ± 5.8***
Lean mass legs (kg)	13.6 ± 1.6	17.3 ± 2.2***

Values presented as mean ± SD. Frequency is presented as n, number and %, percent.

BMD, Bone Mineral Density; BMI, Body Mass Index; HC, hormonal contraceptives; MD, Menstrual dysfunction. MD reported only for participants not using hormonal contraceptives.

*p < 0.05, **p < 0.01, ***p < 0.001.

### Serum IGF-I, IGFBP-1 and Insulin Levels in Athletes and Controls

**Figure 1:**
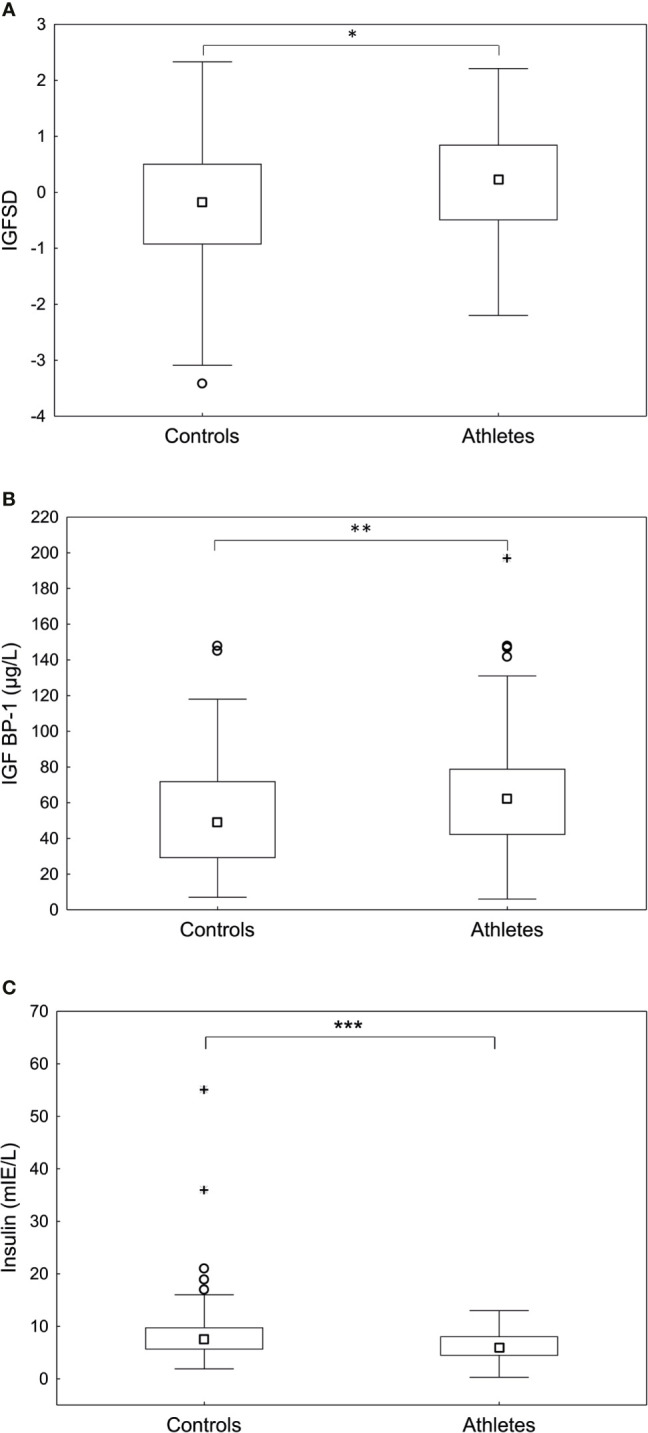
Fasting **(A)** age adjusted insulin-like growth factor-I (IGFSD), **(B)** IGF binding protein- 1 (IGFBP-1) and **(C)** insulin levels in controls and athletes. The athletes demonstrated significantly higher IGFSD and IGFBP-1 and significantly lower insulin levels compared to controls. Box plots showing median, Q25-75, total range, outliers and extremes. *p < 0.05, **p < 0.01, ***p < 0.001.

IGF-I levels in serum were significantly higher in the athletes compared to controls (277.5 ± 85.5 *vs* 249.7 ± 73.3 µg/L, p <0.05). Furthermore, the athletes demonstrated significantly higher levels of IGFSD (p<0.05) and IGFBP-1 (p <0.01), whereas insulin was significantly lower than controls (p <0.001) (**Figures 1A–C**). The frequency of increased fasting serum insulin, indicating insulin resistance, was significantly higher in the controls compared to athletes (n=10 (9%) *vs* n=0 (0%), p <0.01). As expected, IGFBP-1 levels were higher for all hormonal contraceptive users compared to non-hormonal contraceptive users (71.9 ± 35.4 *vs* 50.3 ± 29.4, p<0.001). However, we found no statistically significant interaction between hormonal contraceptive use and IGF-I, IGFSD or IGFBP-1 levels when comparing athletes and controls.

### IGF-I, IGFBP-1 and Insulin Levels in Athletes of Different Sport Categories

**Figure 2:**
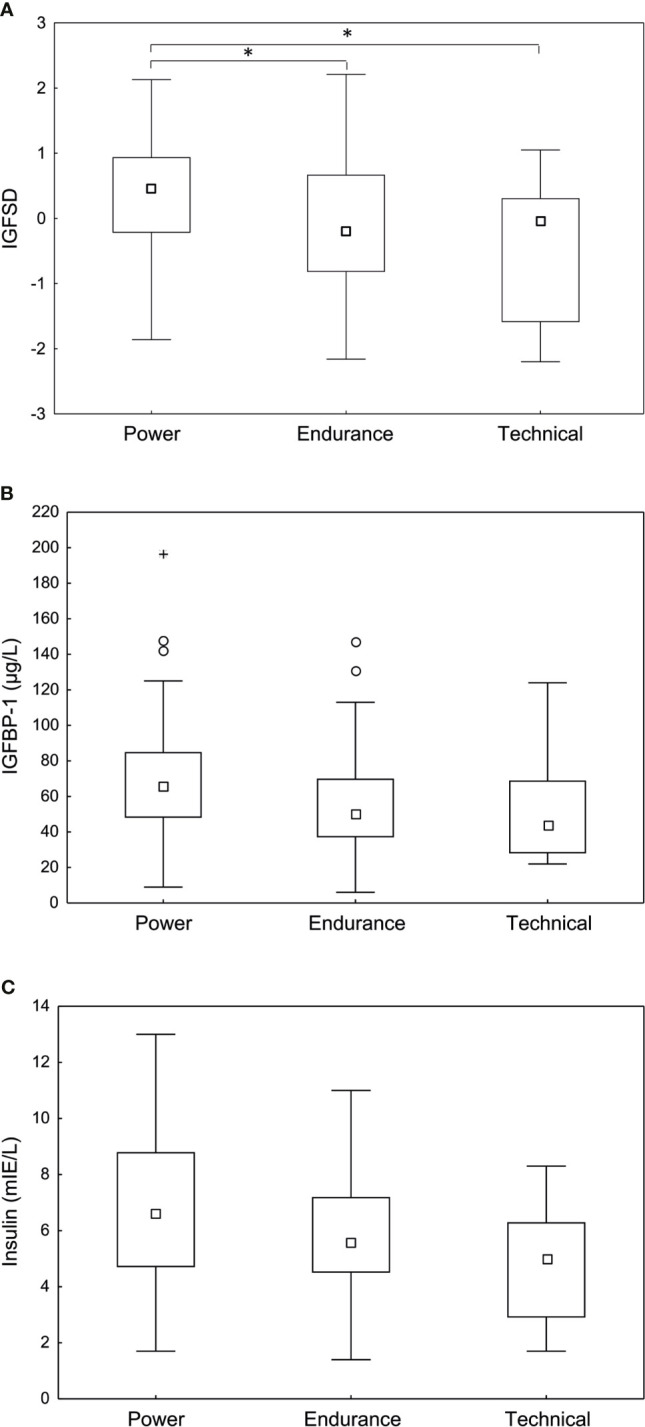
Fasting **(A)** age adjusted insulin-like growth factor-I (IGFSD), **(B)** IGF-I binding protein-1 (IGFBP-1) and **(C)** insulin levels in power, endurance and technical athletes. Power athletes demonstrated significantly higher IGFSD compared to endurance and technical athletes. No significant difference in IGFBP-1 or insulin levels were observed between sport categories. Box plots showing median, Q25-75, total range, outliers and extremes. *p < 0.05.

**Table 3** shows general characteristics and body composition for the athletes depending on sport category (**Table 3**). When comparing sport categories, the power athletes had significantly higher IGF-I compared to technical athletes (293.6±78.4 *vs* 221.5±76.2 (µg/L), p <0.01). Furthermore, power athletes had significantly higher IGFSD (0.37±0.88) compared to both endurance (-0.11±1.18), (p <0.05) and technical athletes -0.44±1.06), (p<0.05) (**Figure 2**). However, IGFBP-1 and insulin levels were comparable between groups (**Figure 2**). No statistically significant interaction was found for hormonal contraceptive use when comparing IGF, IGFSD and IGFBP-1 between sport categories.

**Table 3:** General parameters and body composition in female Olympic athletes of different sport categories power, endurance and technical.

Parameters	Power	Endurance	Technical	P-value
n	62	29	12	
Age	25.0 ± 4.2	24.8 ± 4.3	31.1 ± 9.3	b***c***
BMI	22.1 ± 1.9	21.3 ± 1.4	23.0 ± 2.3	c**
Weight (kg)	65.2 ± 7.5	63.0 ± 7.2	66.3 ± 6.1	
Height (m)	1.72 ± 0.05	1.72 ± 0.07	1.70 ± 0.04	
Exercise time (h/w)	16.5 ± 5.8	20.7 ± 4.5	16.5 ± 4.3	a***c*
HC use, n (%)	23 (37)	13 (45)	4 (33)	
MD, n (%)	7 (18)	6 (38)	2 (25)	
**Body Composition**				
n	42	18	4	
Total BMD (g/cm^2^)	1.28 ± 0.07	1.19 ± 0.07	1.18 ± 0.04	a*** b**
Spinal BMD (g/cm^2^)	1.14 ± 0.1	1.06 ± 0.12	1.02 ± 0.08	a**b*
Z-score	2.04 ± 0.85	0.82 ± 0.85	0.88 ± 0.68	a*** b*
Body fat (%)	19.3 ± 5.3	14.4 ± 4.7	27.5 ± 4.1	a**b** c***
Lean mass total (kg)	49.4 ± 5.9	53.0 ± 4.3	43.3 ± 3.3	a*b* c**
Lean mass legs (kg)	17.3 ± 2.2	17.8 ± 1.8	14.7 ± 1.4	b* c**

Values presented as mean ± SD or median and interquartile range (25th-75th percentile). Frequency is presented as n, number and %, percent.

BMD=Bone Mineral Density, BMD, Bone Mineral Density; BMI, Body Mass Index; HC, hormonal contraceptive; h/w, hours per week; MD, Menstrual dysfunction. MD reported only for participants not using hormonal contraceptives.

a, power vs. endurance; b, power vs. technical; c, endurance vs. technical.

*p < 0.05, **p < 0.01, ***p < 0.001.

### Correlations of IGF-I, IGFBP-1, Insulin and Body Composition

**Figure 3:**
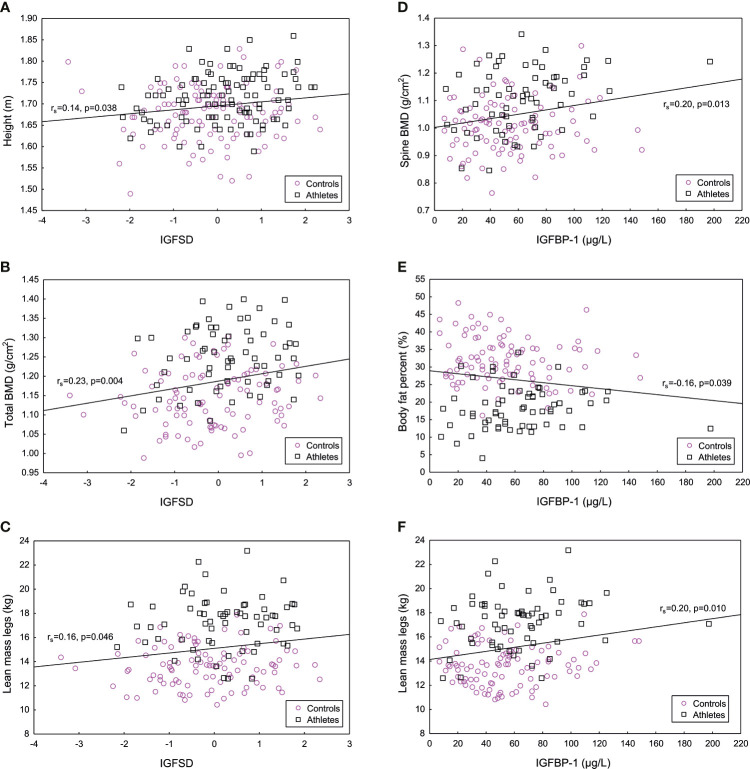
Significant positive correlations between age adjusted insulin-like growth factor-I (IGFSD) and **(A)** height (m), **(B)** total bone mineral density (BMD) (g/cm^2^) and **(C)** lean mass legs (kg), and significant correlations between IGF binding protein 1 (IGFBP-1) and **(D)** bone mineral density (BMD) spine (g/cm^2^), **(E)** body fat percent (%) and **(F)** lean mass legs (kg) for athletes and controls combined. Magenta symbol representing controls and black symbol representing female Olympic athletes.

In the total population of athletes and controls, significant, positive correlations were found between IGFSD and height, total BMD and lean mass legs (kg) (**Figure 3**), as well as Z-score (r_s_=0.23, p <0.01). IGFBP-1 correlated positively to BMD spine, lean mass total (kg) (r_s_=0.20, p <0.05) and lean mass legs (kg) and negatively correlated to fat percent (**Figure 3**). Most of these results remained in the subgroup of participants not using hormonal contraception (data not shown). Insulin correlated positively to fat percent (r_s_=0.28, p <0.001) and negatively to total BMD (r_s_= -0.20, p <0.05), Z-score (r_s_= -0.27, p <0.001) and lean mass total (kg) (r_s_=-0.20, p <0.01).

In the subgroup of athletes, there were significant positive correlations between IGF-I and height (r_s_ = 0.21, p <0.05), as well as between IGFSD and height (r_s_=0.21, p<0.05). Furthermore, IGFBP-1 correlated positively to total BMD (r_s_=0.28, p<0.05) and BMD spine (r_s_=0.28, p<0.05). For the controls, no significant correlations were found between IGF-I, IGFSD, IGFBP-1 and body composition, whereas insulin correlated negatively to Z-score (r_s_=-0.26, p=0.01).

### Correlations of IGF-I and IGFBP-1 With Physical Fitness Test in Female Athletes

**Figure 4:**
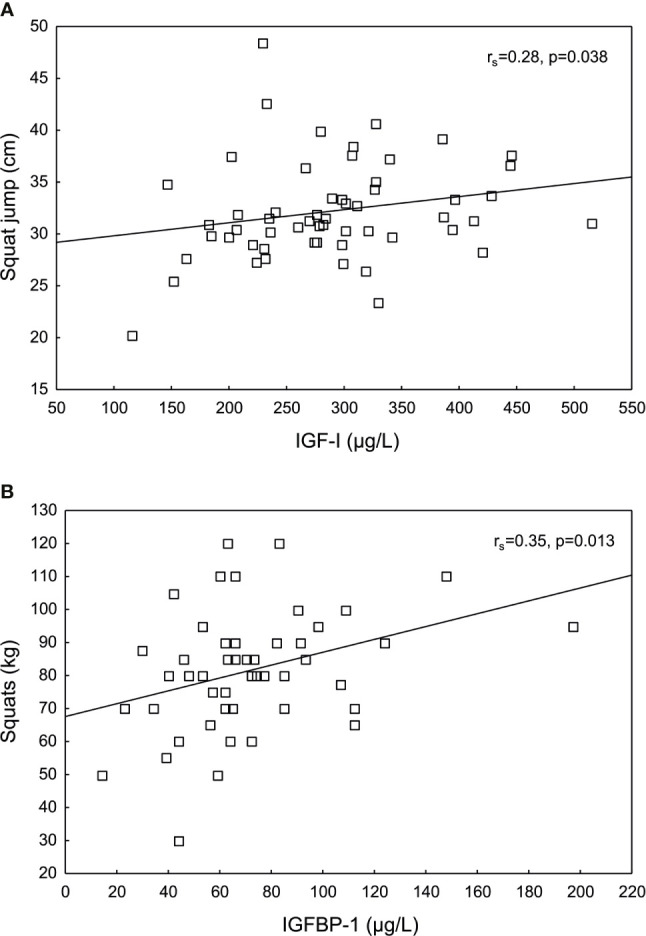
Significant positive correlations between **(A)** insulin-like growth factor I (IGF-I) and squat jump (SJ), and between **(B)** IGF binding protein 1 (IGFBP-1) and squats in female Olympic athletes.

IGF-I levels correlated positively to SJ, whereas IGFBP-1 levels correlated positively to squats (**Figure 4**). No other significant correlations were found between IGF-I, IGFSD, IGFBP-1 or insulin and the physical fitness tests, data not shown.

### Correlations Between IGF-I, IGFBP-1, and Insulin

In all participants, significant positive correlations were found between insulin and IGF-I (r_s_=0.24, p=0.0004) and IGFSD (r_s_=0.19, p=0.005), respectively. Furthermore, insulin correlated negatively to IGFBP-1 (r_s_=-0.21, p=0.002). In the subgroup of athletes, insulin correlated positively to IGF-I (r_s_=0.40, p=0.00005) and IGFSD (r_s_=0, 34, p=0.0004). In the control group, insulin correlated positively to IGF-I (r_s_=0.19, p=0.04) and negatively to IGFBP-1 (r_s_=-0.19, p=0.04).

### Multiple Regression Analysis

The multiple regression analysis showed that the strongest factor explaining the variance in IGF-I levels was insulin levels (β=0.31, p <0.001) followed by group (athletes and controls), (β=-0.20, p <0.01) and height (β=0.14, p <0.05) together accounting for 12% of the variance in IGF-I levels.

## Discussion

In support of our hypothesis, female Olympic athletes had significantly higher IGF-I, IGFSD and IGFBP-1 levels compared to controls, and the highest levels were in power athletes. Furthermore, endogenous levels of IGF-I and IGFBP-1 were associated with body composition and correlated positively with physical fitness in the athletes.

In agreement with our findings, higher calculated IGF-I reference ranges were reported in 58 female volleyball players compared to that of a female reference population (18). However, Snow et al. (20) found no difference in IGF-I levels between 23 female gymnasts and middle- and long-distance runners and 13 controls. Similarly, another study showed no differences between 29 athletes participating in various sports and 11 controls (21). When comparing sport categories, Snow et al. (20) reported higher serum IGF-I levels in gymnasts compared to runners, which is in agreement with our findings of the highest IGFSD in power athletes. However, in a large cross-sectional study by Healy et al. (22), athletes in endurance sports such as swimmers and cross-country skiers demonstrated the highest postcompetition serum IGF-I levels.

IGF-I is known to have anabolic effects on adult bone remodeling (37) and to stimulate protein synthesis in human skeletal muscle (38). The existing literature on the relationship between circulating IGF-I and body composition in female athletes is very limited. Healy et al. (22) showed no significant association between IGF-I levels and body fat, measured by bio-impedance in 92 female athletes. However, in another study including female athletes, IGF-I correlated positively with body weight (24). Furthermore, in the study by Snow et al. (20), significant positive correlations between IGF-I, BMD and lean mass were reported. In agreement, we found positive, but weak, correlations between IGFSD and BMD, as well as lean body mass in the combined group of athletes and controls. Similar correlations were found for IGFBP-1, and in addition a significant negative correlation with fat percent.

To our knowledge, this study is the first to investigate endogenous IGF-I in relation to athletic performance in female elite athletes. Here, we demonstrate significant positive associations between IGF-I and IGFBP-1 and SJ and squats, respectively. Our findings may support an anabolic role of endogenous IGF-I in female athletes in power sports. The positive association with higher IGFBP-1 levels could hypothetically be related to increased insulin sensitivity and or increased capillary density and blood flow in the muscles. However, since only two out of several physical fitness tests were significant, these results should be interpreted with caution. A few other studies have investigated associations between endogenous IGF variables and fitness in adolescent and adult women, without showing consistent results (25–27). The majority of evidence suggesting a positive effect of the GH/IGF-I systems on physical fitness is based on exogenous GH and IGF-I given to healthy populations (3, 9, 17). Although these substances are classified as doping agents, the physical performance enhancing effects of GH/IGF supplementation in healthy adults are debated (3, 7, 8).

The underlying mechanisms for our observed findings of differences in endogenous IGF-I levels between athletes and controls and between sport categories are not known. Since our study is of cross-sectional design, we cannot make any assumption of causality. However, both adaptive responses of the GH/IGF-I system to exercise, nutritional status and a genetic predisposition could hypothetically be possible explanations. Studies investigating acute and chronic effects of exercise on circulating IGF-I levels have generated varying results (1, 18, 19, 24, 39–41). Hence, it is still unclear how exercise influences circulating IGF-I levels. Considering IGFBP-1, exercise has been associated with increased serum IGFBP-1 levels (1) possibly due to an increase in HIF-1a (15).

In addition, it is well known that IGF-I increases in response to food intake especially amino acids/proteins while IGFBP-1 levels decrease. However, this is not likely to have influenced our results since all blood samples were taken in a fasting state. Furthermore, long term energy deficiency is known to reduce IGF-I production, resulting in lower serum and tissue levels of IGF-I (37, 42), and reduced insulin and increased IGFBP-1 (2, 37, 42, 43). Chronic energy deficiency is common among elite athletes, both in females and males (44, 45). Although evaluation of nutritional intake and energy balance was not the focus of the present study, energy deficiency it is not likely the reason for the elevated IGFBP-1 and lower insulin levels among athletes, since they also presented with higher IGF-I levels and IGFSD compared to controls. A more reasonable explanation for our findings is an increased insulin sensitivity in the athletes compared to the controls. This is supported by the well- known associations between insulin sensitivity and high levels of IGFBP-1 and low levels of insulin (2). Furthermore, the multiple regression analysis showed that the strongest factor explaining the variance in IGF-I levels were insulin levels.

There is strong evidence of a genetic influence on IGF-I and IGFBP-1 levels, accounting for 63% and 36% of the variance, respectively (46). Our finding of a positive correlation between IGF-I and height may indicate that the higher IGF-I levels in athletes are genetically determined. The multiple regression analysis showed that height together with group (athletes and controls) were the second strongest factors after insulin determining IGF-I levels. Furthermore, in athletes, IGF-I levels have a low intra-individual variability (14-16%), being stable despite different training condition, age and gender (47). Hypothetically, the higher IGF-I and IGFSD in the athletes, especially those participating in power sports, reflect a more anabolic hormonal constitution due to a genetic predisposition.

Certain limitations of the present investigation should be addressed. It should be mentioned that the reference values of age adjusted IGF-I are based on a limited number of women in each age category. Furthermore, hormonal changes during the menstrual cycle and hormonal contraceptive use may have influenced the results. We found it too challenging to consider the menstrual cycle in the athletes due to logistical reasons. However, since the variations in IGF-I levels during the menstrual cycle have been described to be non-existing (48) or modest (49, 50), and unchanged levels have been reported for IGFBP-1 (48, 49), we believe that the results presented here are independent of the menstrual cycle. Furthermore, menstrual dysfunction is not likely the reason for elevated IGF variables in the athletes since previous studies have demonstrated no difference in total IGF-I levels in female athletes with or without menstrual dysfunction (30, 43).

A large percentage of the female Olympic athletes used hormonal contraception. The effects of hormonal contraceptives on the GH/IGF-I system is complex and are dependent on type of estrogen, route of administration, duration of treatment and dose (51). In this study, we found no significant difference in IGF-I or IGFSD between groups depending on hormonal contraceptive use. In agreement, two previous studies including female athletes found no significant effect of oral contraceptive use on serum IGF-I levels (43, 52). However, as previously described (30, 43), IGFBP-I levels were higher among hormonal contraceptive users.

Even though exogenous GH and IGF-I are classified as doping agents, we have not evaluated the potential misuse of these substances in our study population. However, all female athletes that participated in this study were screened for anabolic androgenic steroids. Furthermore, they are regularly included in doping control tests according to the WADA code. Therefore, we find it unlikely that our results are explained by doping. Further limitations of the study are the relative low number of athletes that performed physical fitness tests, as well as the heterogeneity of sports performed by the included athletes.

In conclusion, our results suggest that athletes have a more anabolic hormonal constitution compared to untrained controls, hypothetically due to a genetic predisposition. Furthermore, our results suggest that endogenous IGF-I and IGFBP-1 may be of importance for body composition and physical performance in elite female athletes. However, further studies are warranted to elucidate underlying mechanisms and potential causality behind the associations.

## Data Availability Statement

The raw data supporting the conclusions of this article will be made available by the authors, without undue reservation.

## Ethics Statement

The studies involving human participants were reviewed and approved by the Regional Ethics Committee, Stockholm (EPN 2011/1426-32). The patients/participants provided their written informed consent to participate in this study.

## Author Contributions

ALH, BB, and EE were involved in the concept/design of the study. ALH and EE were responsible for the acquisition of data and in collaboration with AH and KB also the data analysis. EE, AH, BB, KB, and ALH were involved in the manuscript preparation, critical revision of the article and approval of the article. All authors listed met the conditions required for full authorship. All authors contributed to the article and approved the submitted version.

## Funding

The study was financed by grants from the Swedish Research Council (2017-02051), the Swedish Research Council for Sport Science and the Clinical Scientist Training Programme and the Clinical Research Internship Programme from the Karolinska Institutet.

## Conflict of Interest

BB is the medical director for the Swedish Olympic Committee (SOC) and ALH is medical adviser to the SOC, the International Association of Athletic Federation (IAAF) and the International Olympic Committee (IOC).

The remaining authors declare that the research was conducted in the absence of any commercial or financial relationships that could be construed as a potential conflict of interest.

## Publisher’s Note

All claims expressed in this article are solely those of the authors and do not necessarily represent those of their affiliated organizations, or those of the publisher, the editors and the reviewers. Any product that may be evaluated in this article, or claim that may be made by its manufacturer, is not guaranteed or endorsed by the publisher.
